# Accuracy of smartphone photographs for detecting active carious lesions in orthodontic patients

**DOI:** 10.1590/1807-3107bor-2025.vol39.069

**Published:** 2025-09-08

**Authors:** Ricardo Kenji TAKAHAMA, Bianca Schlesener DETTMER, Larissa Lemos NAGIPE, Patricia Kolling MARQUEZAN, Luana Severo ALVES, Júlio Eduardo do Amaral ZENKNER

**Affiliations:** (a) Universidade Federal de Santa Maria –UFSM, Department of Stomatology, Santa Maria, RS, Brazil.; (b) Universidade Federal de Santa Maria – UFSM, Department of Microbiology and Parasitology, Santa Maria, RS, Brazil.; (c) Universidade Federal de Santa Maria – UFSM, Department of Restorative Dentistry, Santa Maria, RS, Brazil.

**Keywords:** Dental Caries, Dental Caries Activity Tests, Telediagnostics, Photography, Dental

## Abstract

Advancements in digital media have driven the study and use of photographic records as a diagnostic method for carious lesions, with smartphone images being widely utilized across various health fields. This study aimed to evaluate the diagnostic accuracy of smartphone photography for detecting active caries in orthodontic patients. The sample comprised 100 individuals of both sexes, aged 11 to 46 years, who were undergoing fixed orthodontic treatment. Following professional tooth cleaning and drying, patients underwent a visual-tactile clinical examination for caries detection, which served as the gold standard. Digital photographs were then taken using a smartphone camera in five views: frontal, right and left lateral, and upper and lower occlusal. The diagnostic parameters—sensitivity, specificity, accuracy, positive predictive value (PV+), and negative predictive value (PV-)—were calculated for the photographic method relative to the clinical examination. The prevalence of active caries in the sample was 74%. The smartphone photographs correctly identified 66 of the 74 individuals with active caries according to the gold standard. However, only 4 of the 26 sound patients were correctly identified. These findings correspond to the following diagnostic parameters: sensitivity, 89%; specificity, 15%; accuracy, 70%; PV+, 75%; and PV-, 33%. In conclusion, the photographic method demonstrated high sensitivity and satisfactory accuracy in detecting caries in patients undergoing fixed orthodontic therapy. However, the low specificity observed suggests a tendency to overdiagnose sound teeth.

## Introduction

At least one-third of the global population is affected by caries disease.^1,2^ It is the most common adverse effect in patients undergoing fixed orthodontic treatment.^3^ These patients have a high incidence and prevalence of white spot lesions,^4^ and the longer the duration of orthodontic treatment, the greater the prevalence of carious lesions.^5^ This is due to difficulties in maintaining oral hygiene around orthodontic wires, brackets, and bands, as well as the limiting effect of fixed appliances on the self-cleaning mechanism of saliva and oral musculature.^4^ Consequently, plaque accumulates on tooth surfaces, leading to a decrease in pH and subsequent demineralization of dental hard tissues.^4,6^

Although the visual-tactile examination remains the most commonly used method for detecting carious lesions,^7^ many studies investigating the association between fixed orthodontic appliances and dental caries have relied on photographs of dental arches for caries detection,^8-11^ primarily those taken for orthodontic documentation. Compared to digital cameras, smartphones offer advantages such as easier image storage and sharing, affordability, portability, and user friendliness, while still producing satisfactory images with minimal training.^12-14^ The use of smartphone photography has been effective in various health fields,^15^ and its application in large-scale dental screening is particularly relevant, since it represents an economical and valid alternative as a screening tool. Additionally, mobile technologies and digital photography can help mitigate the challenges posed by geographical inaccessibility and pandemic-related dental service disruptions.^16^

Several studies have assessed the diagnostic accuracy of smartphone photographs for caries detection using clinical examination as the gold standard.^17-20^ In a sample of 100 participants, smartphone photographs demonstrated sensitivity and specificity values ranging from 60% to 63% and 96% to 99%, respectively, and were effective in distinguishing sound surfaces from extensive lesions (75–100% sensitivity, > 83% specificity).^17^ However, a study comparing two smartphone models on deciduous molars of 15 children reported low sensitivity (< 50%) for detecting initial and moderate carious lesions.^18^ Two other studies assessed smartphone photographs taken by family members: one involving 42 children reported high specificity (> 95%) but low sensitivity (44–88.4%),^19^ while the other, with 43 patients, showed good sensitivity (74%) and high specificity (99.1%).^20^

Despite these findings, no studies have evaluated the diagnostic accuracy of smartphone photography for detecting caries lesions in patients with fixed orthodontic appliances. If proven accurate, this method could aid in caries detection, longitudinal follow-up, and screening. Therefore, this study aimed to assess the diagnostic accuracy of smartphone photography for detecting active caries in orthodontic patients. The null hypothesis was that caries detection based on smartphone photography would yield results similar to those of clinical examination.

## Methods

This study was reported according to the Standards for the Reporting of Diagnostic Accuracy Studies (STARD).^20^

### Study design and sample

This observational cross-sectional study included a convenience sample of patients undergoing orthodontic treatment in a specialization course in Orthodontics and at the dental service of the Military Air Force of Santa Maria, southern Brazil. Individuals of both sexes using any type of fixed appliance, regardless of usage duration, were invited to participate. No exclusion criteria were applied.

### Reproducibility

Clinical examinations were conducted by two examiners (LLN and PKM), both trained and calibrated for caries assessment by a reference examiner. LLN examined 38 participants, while PKM examined 62. A single examiner (PKM) evaluated the photographs. Intra-examiner reproducibility for clinical examinations was assessed by performing repeated assessments on 15 participants, with a minimum interval of seven days. The unweighted kappa values were 0.70 for LLN and 0.85 for PKM, while inter-examiner agreement yielded a kappa value of 0.70. Intra-examiner reproducibility for photographic examinations was also evaluated by re-examining images from 11 patients, yielding a kappa value of 0.94.

### Data collection

Patients first underwent tooth cleaning with a toothbrush and prophylactic paste. Clinical examinations were conducted in a dental unit using a flat clinical mirror (No. 5) and a World Health Organization probe. Following this, the teeth were isolated with cotton rolls and air-dried for five seconds using an air-water syringe. Caries assessment included an evaluation of all dental surfaces, which were classified based on their clinical characteristics—color, brightness, and surface texture—as follows:

- Inactive non-cavitated lesions: Shiny surface areas with or without varying degrees of brownish discoloration.
- Active non-cavitated lesions: Opaque enamel with a dull-whitish surface.
- Inactive cavitated lesions: Localized surface destruction with arrested characteristics (shiny, hard surfaces with varying degrees of brownish discoloration).
- Active cavitated lesions: Localized surface destruction with active characteristics (dull-whitish enamel and soft dentin with a light brown color).^22^

Additionally, the presence of restored or missing teeth, crowns, calculus, dental fluorosis, and dentin dark shadows was recorded.

After the clinical examination, a trained professional captured five standardized photographs of each patient—frontal, left lateral, right lateral, maxillary occlusal, and mandibular occlusal views—following the American Board of Orthodontics requirements for intraoral photographs. Photographs were taken in a dental unit, with the patient in a supine position. Cheek retractors were used to aid imaging (two C-shaped retractors for frontal, maxillary occlusal, and mandibular occlusal views, and one C-shaped plus one V-shaped retractor for left and right lateral views). A double-sided mirror was used for intraoral occlusal views. Before imaging, the teeth were dried using an air-water syringe. An iPhone 8 (Apple, Cupertino, USA) was used to capture the images from a standardized distance of 10–15 centimeters from the dental arches. The flash was not used; however, brightness adjustments and zooming (1-2x) were applied when necessary. Additionally, a selfie ring light was attached and set to intensity 4 to optimize lighting conditions. Finally, each patient completed a self-administered questionnaire covering sociodemographic and behavioral aspects for sample characterization. Underage participants received assistance from their parents or legal guardians.

### Photographic analysis

Dental caries detection using the intraoral photographic method followed the same criteria as the clinical examination. Data were recorded on a clinical sheet identical to the one used for the clinical assessment. All surfaces from the first right molar to the first left molar were evaluated in both arches. Photographic evaluations were conducted using the same notebook, with a standardized screen configuration and without image magnification. The examiner was blinded to the clinical diagnosis, since photographic assessments were performed at least 15 days after the clinical examination, with no identifying information provided in the images.

### Data analysis

The prevalence of caries activity was defined as the percentage of individuals with at least one active carious lesion, whether non-cavitated or cavitated. The diagnostic parameters—sensitivity, specificity, positive predictive value, negative predictive value, and accuracy—were calculated for the photographic method, with the clinical examination serving as the gold standard.

### Ethical aspects

The study protocol was approved by the Ethics Committee of the Federal University of Santa Maria (CAAE 10325518.9.0000.5346). Patients or their legal guardians provided informed consent. Each participant received a report on their oral health status.

## Results

As shown in Table 1, the sample comprised 100 individuals, predominantly young adults (mean ± standard deviation [SD] age: 27 ± 16 years) and females (66%). The prevalence of caries activity was 74% (n = 74 patients). On average (± SD), these individuals had 3.5 (± 3.6) active lesions, with a range of 0 to 16 (median: 2, interquartile range: 0–6).

**Table 1:** Sample description.

Variables	(n = 100)
Sex, m:f	34:66
Age, mean (±SD)	27 (±16)
Active lesions	
Prevalence	74%
Mean (± SD)	3.5 (±3.6)
Median (p25; p75)	2 (0; 6)
Range (min-max)	0-16
Family income, n (%)	
< 1 BWM	4 (4)
1–3 BMW	50 (50)
> 3 BMW	46 (46)
Mother’s education level*, n (%)	
≤ Elementary school	44 (44.9)
High school	32 (32.6)
College	22 (22.4)
Tooth brushing frequency*, n (%)	
≤ twice/day	22 (22.7)
> twice/day	75 (77.3)
Use of dental floss*, n (%)	
Yes	81 (82.6)
No	17 (17.4)

BMW: Brazilian Minimum Wage, m: male, f: female. *Missing data.

Table 2 presents the diagnostic parameters derived from the comparison between the clinical examination and digital photographs taken with a smartphone for detecting caries-active orthodontic patients. The digital photographic method correctly identified 66 out of 74 individuals with active caries, according to the gold standard. However, only 4 out of 26 sound patients were correctly classified. These results correspond to the following diagnostic parameters: sensitivity, 89%; specificity, 15%; accuracy, 70%; positive predictive value, 75%; and negative predictive value, 33%.

**Table 2:** Diagnostic parameters of smartphone-based digital photographs for detecting caries-active orthodontic patients (n = 100).

Variables	Clinical examination (gold standard)		Total
	Present	Absent	
Photographic method			
Present	66 (TP)	22 (FP)	88
Absent	8 (FN)	4 (TN)	12
Total	74	26	100

TP: true positives; FP: false positives; TN: true negatives; FN: false negatives.

## Discussion

This study assessed the diagnostic accuracy of the photographic method using a smartphone camera for detecting active caries in patients undergoing fixed orthodontic therapy. Among these individuals, the method demonstrated sufficient accuracy (70%) and high sensitivity (89%) for detecting active carious lesions. To the best of our knowledge, this is the first study to evaluate the accuracy of smartphone-based photographic detection specifically in orthodontic patients.

Communication technology has the potential to improve oral health at the community level and promote universal coverage. A study conducted among 12-year-old schoolchildren in a rural Indian setting demonstrated that smartphone-based video-graphic caries assessment provided an economical diagnostic tool that could help reduce oral health disparities.^23^ Teledentistry, which relies on photographic technology and digital clinical records, facilitates remote consultations by enabling the exchange of clinical images.^24^ Notably, implementing a teledentistry model using a smartphone camera resulted in an estimated savings of AUD 85 million compared to conventional face-to-face dental screening in Australia.^25^ These findings highlight the potential application of smartphone-based photography for caries screening, thus reinforcing its feasibility as a cost-effective tool in oral healthcare.

A high prevalence of caries activity was observed among the evaluated patients, which aligns with previous literature on this topic. Pinto et al.^26^ reported that individuals undergoing fixed orthodontic treatment were nearly 10 times more likely to develop at least one active carious lesion than those without a fixed appliance over a one-year period. Additionally, the same research group demonstrated a progressive increase in caries prevalence with longer orthodontic treatment duration. Among adolescents and young adults, the proportion of patients with active carious lesions differed significantly across treatment groups: 1.5% in untreated individuals, 27.7% after one year of treatment, and 72.3% after two to three years of treatment.^5^ These findings underscore the critical need for continuous monitoring of orthodontic patients for caries development throughout the treatment.

In this study, smartphone photographs corretly identified most patients with caries activity (66 out of 74), yielding a high sensitivity (89%). These findings indicate that digital photographs were effective in detecting active caries in patients with fixed orthodontic appliances. Similar results had been reported in studies comparing photographic diagnosis using smartphones with clinical examination. Kohara et al.^18^ evaluated two smartphone models for detecting caries at different stages on the occlusal surfaces of deciduous molars in 15 children. Sensitivity was 75%–100% for extensive lesions, but lower (53.1%) for initial and moderate lesions. Zotti et al.^20^also found a good sensitivity value (74%) when assessing remote caries diagnosis using five intraoral photographic views (frontal, right and left lateral, upper and lower occlusal) of 43 young adults (22-38 years old) taken at home by a family member.

These studies suggest that smartphone-based photographic diagnosis can be effective, particularly for detecting more advanced carious lesions. On the other hand, Estai et al.^17^ and Azimi et al.^19^ reported lower sensitivity values than those found in our study. Estai et al.^17^ found sensitivity values of 60–63% in a sample of 100 patients of varying ages, where smartphone photographs were taken without mirrors or cheek retractors. Azimi et al.^19^reported a sensitivity range of 44%–88.4% in a study of 42 children under four years old, where an application operated by parents was used to detect early childhood caries without prior tooth brushing, drying, or the use of mirrors. These methodological differences likely contributed to the lower sensitivity observed in their findings. Considering these aspects, it is plausible that the use of cheek retractors, mirrors, and pre-photography tooth cleaning and drying in our study contributed to the higher sensitivity of the photographic method compared to the studies by Estai et al.^17^ and Azimi et al.^19^

Regarding specificity, only 4 out of 26 sound patients were correctly identified using the photographic method, resulting in a low specificity (15%). This finding differs from previously cited studies, which reported notably higher specificity values (Estai et al.,^17^ 96–99%; Zotti et al.,^20^ 99.1%; Azimi et al.,^19^ > 95%; and Kohara et al.^18^: > 83%). It is important to note that none of these studies focused on orthodontic patients, which may have contributed to the differences in specificity.

Given that our study reported the lowest specificity among these investigations, several possible explanations must be considered. One potential reason is that some lesions classified as inactive during the clinical examination may have been identified as active in the photographic assessment due to the absence of tactile feedback to evaluate surface roughness. Additionally, many patients in our study had dental fluorosis, which may have been misdiagnosed as active caries on photographic examination. Another contributing factor could be the presence of composite resin around brackets, which may have been mistaken for active carious lesions in some cases. Finally, our statistical analysis was conducted at the patient level rather than the tooth level, based on the premise that once a patient exhibits caries activity, treatment is required, regardless of the number of lesions. In this context, a single misdiagnosed tooth in a sound patient classifies the entire patient as caries-active in the analysis, thereby reducing specificity. The low specificity observed in our study suggests that the photographic method may overdiagnose sound patients.

In the age of technology and increasing global connectivity, where smartphone ownership is widespread, smartphone integration into dental services is expected to grow due to its digital imaging capabilities, computational power, sharing ability, affordability, and secure cloud storage.^17^ Considering these advantages, smartphone-based photography could serve as a screening tool to identify patients who require a clinical examination. To properly discuss this aspect, it is essential to distinguish between screening tests and confirmatory tests.^27^ Screening tests are used at the population level to identify individuals who may have a particular disease, even if they are asymptomatic, but such tests are not definitive. In contrast, confirmatory tests are typically performed after a screening test to establish a diagnosis. Given this distinction, screening tests must prioritize high sensitivity to minimize false negatives, and thus ensure that affected individuals are identified. However, they may admissibly exhibit lower specificity for detecting the condition under investigation, thus leading to some false positives.

When a screening test suggests the possible presence of a disease, additional diagnostic tests are typically required. Confirmatory tests must be highly specific to minimize false positives, thus providing a more accurate and definitive diagnosis to resolve any uncertainty from the screening results. However, confirmatory tests are often more expensive, time-consuming, or invasive, making their routine application neither ethical nor feasible.^28^

Considering these aspects, smartphone-based photography could serve as a screening tool for the early detection of carious lesions in orthodontic patients, while traditional clinical oral examination would act as the confirmatory test to assess caries activity and determine the need for treatment. Importantly, the low specificity of the photographic method does not pose a risk, since patients would still undergo a clinical evaluation to confirm their caries-active (requiring treatment) or caries-inactive (not requiring treatment) status. This classification is particularly relevant, since most lesions detected were white spot lesions—caries in a reversible stage—where non-invasive treatment is indicated.^29^ Teleconsultation gained widespread popularity in medicine during the COVID-19 pandemic, and it is expected to become increasingly relevant in dentistry.^30^ In this context, further studies are needed to evaluate the accuracy of photographs taken by family members of orthodontic patients, and thus expand the potential applications of smartphone-based caries screening.

One of the key strengths of this study is its pioneering nature, since it is the first to evaluate the diagnostic accuracy of the smartphone-based photographic method for detecting carious lesions in patients with fixed orthodontic appliances. Additional strengths include the use of trained and calibrated examiners for caries assessment, as well as the standardized photographic protocol, which adhered to American Board of Orthodontics requirements for intraoral photographs and included tooth cleaning and drying prior to imaging. A notable limitation of this study is the imbalance between caries-active and sound patients, despite the reasonable sample size. This discrepancy may have influenced the diagnostic parameters, particularly specificity.

## Conclusion

This study demonstrated that smartphone-based photographic examination achieved satisfactory accuracy (70%) in detecting caries-active patients during fixed orthodontic therapy, with high sensitivity (89%). However, the low specificity (15%) suggests that this photographic method may overdiagnose sound patients within the study sample.

## Data Availability

The datasets generated and analyzed during the current study are not publicly available because the authors do not hold full rights over the data and therefore cannot commit to making them available in the future. However, the data are available from the corresponding author upon reasonable request.
